# Mussel-Inspired and Bioclickable Peptide Engineered Surface to Combat Thrombosis and Infection

**DOI:** 10.34133/2022/9780879

**Published:** 2022-04-14

**Authors:** Xiaohui Mou, Hongbo Zhang, Hua Qiu, Wentai Zhang, Ying Wang, Kaiqin Xiong, Nan Huang, Hélder A. Santos, Zhilu Yang

**Affiliations:** ^1^Affiliated Dongguan Hospital, Southern Medical University, Dongguan, Guangdong 523059, China; ^2^Guangdong Provincial Key Laboratory of Shock and Microcirculation, Guangzhou, Guangdong 510080, China; ^3^Key Laboratory of Advanced Technologies of Materials, Ministry of Education, School of Materials Science and Engineering, Southwest Jiaotong University, Chengdu 610031, China; ^4^Pharmaceutical Sciences Laboratory, Åbo Akademi University, Turku Biosciences Center, University of Turku and Åbo Akademi University, 20520 Turku, Finland; ^5^State Key Laboratory of Molecular Engineering of Polymers, Fudan University, Shanghai 200438, China; ^6^Department of Biomedical Engineering and W.J. Kolff Institute for Biomedical Engineering and Materials Science, University Medical Center Groningen/University of Groningen, Ant. Deusinglaan 1, 9713 AV Groningen, Netherlands; ^7^Drug Research Program, Division of Pharmaceutical Chemistry and Technology, Faculty of Pharmacy, University of Helsinki, FI-00014 Helsinki, Finland

## Abstract

Thrombosis and infections are the two major complications associated with extracorporeal circuits and indwelling medical devices, leading to significant mortality in clinic. To address this issue, here, we report a biomimetic surface engineering strategy by the integration of mussel-inspired adhesive peptide, with bio-orthogonal click chemistry, to tailor the surface functionalities of tubing and catheters. Inspired by mussel adhesive foot protein, a bioclickable peptide mimic (DOPA)_4_-azide-based structure is designed and grafted on an aminated tubing robustly based on catechol-amine chemistry. Then, the dibenzylcyclooctyne (DBCO) modified nitric oxide generating species of 1,4,7,10-tetraazacyclododecane-1,4,7,10-tetraacetic acid (DOTA) chelated copper ions and the DBCO-modified antimicrobial peptide (DBCO-AMP) are clicked onto the grafted surfaces via bio-orthogonal reaction. The combination of the robustly grafted AMP and Cu-DOTA endows the modified tubing with durable antimicrobial properties and ability in long-term catalytically generating NO from endogenous s-nitrosothiols to resist adhesion/activation of platelets, thus preventing the formation of thrombosis. Overall, this biomimetic surface engineering technology provides a promising solution for multicomponent surface functionalization and the surface bioengineering of biomedical devices with enhanced clinical performance.

## 1. Introduction

The commonly used indwelling catheters and external circuits in medical scenarios (i.e., ventricular assist devices, pacemakers, artificial hearts, hemodialysis, and cardioverter defibrillators) have been considered as the “lifeline” of patients [1, 2]. However, thrombosis and infections of extracorporeal circuits and indwelling medical devices often cause device failure [3, 4] along with serious complications, including for example catheter-related thrombosis [5], catheter-related bloodstream infection [6], and deep phlebitis [7]. Specifically, the formation of bacterial biofilm and thrombus on the surfaces of such types of blood-contacting devices is the major difficulty in the long-term treatment.

In traditional clinical practices, coadministration of antibiotics and anticoagulant drugs has been widely applied to prevent thrombosis and infection. However, this strategy can lead to various clinical complications, e.g., antibiotic resistance [8], bleeding [9], thrombocytopenia [10], and allergic reaction [11]. Accordingly, endowing the implanted materials or devices with multifunctionalities to combat thrombosis and infection has attracted increasing attentions in recent years [12, 13]. Among these strategies, grafting bioactive molecules, such as anticoagulant molecules [14], antibiotics [15], and peptide [16], which provide localized bioactivity on the surface of device, has been considered as one of the most effective methods for suppressing thrombosis and infections. However, the addressing of the two complications simultaneously is still a formidable challenge as yet. Strategies that “learn from Nature” [17] may be a promising way to solve such problems.

In circulation, the natural blood vessels show remarkable anticoagulant and antibacterial properties, which are associated with the microenvironment of the blood vessels and their biological activities [18]. Antimicrobial peptide (i.e., AMP), being widely distributed in plasma, directly kills bacteria when wound occurs, which is a crucial part of the immune system [19]. Compared with antibiotics, AMP has a wide range of antibacterial mechanisms and does not cause bacterial resistance [20]. In addition, endothelium exhibits a crucial effect in maintaining vascular homeostasis through releasing a kind of factors (e.g., nitric oxide (NO)) [21–23]. Endothelial cells continuously and stably release NO into blood microenvironment, which has a notable suppressive effect on platelet activation adhesion and thrombosis [24]. Specially, the 1,4,7, 10-tetraazocyclododecane-1,4,7, 10-tetraacetic acid chelated copper ion (i.e., Cu-DOTA) is widely used as a stable and effective biologically active catalyst to decompose endogenous S-nitrosothiols (RSNOs) into NO [25, 26]. Therefore, blood contact device with NO-generating function and surface engineered antimicrobial peptides may provide a highly simulated vascular microenvironment to prevent thrombosis and bacterial infection. However, the inevitably consumed active groups (e.g., -COOH, -NH_2_, and -SH) of such biomolecules after chemical immobilization would result in a progressive loss of bioactivity as reported [27]. Besides, the current chemical modification methods suffer from tedious reaction processes and complex surface treatment technologies, compromising controllability, maneuverability, and reproducibility of surface bioactivity. Considering these issues, click chemistry, a concept founded in 2001 [28], is considered as an effective tool to address these issues to some extent [29]. In addition, bio-orthogonal click chemistry, a new click chemistry method [30], e.g., the dibenzylcyclooctyne-azide (N_3_-DBCO) cycloaddition chemistry, demonstrates advantages in rapidity, thoroughness, and specificity [31, 32]. The high fidelity of these reactions in the face of a wide range of functional groups allows them to better maintain the biological activity of biomolecules.

**Figure 1:**
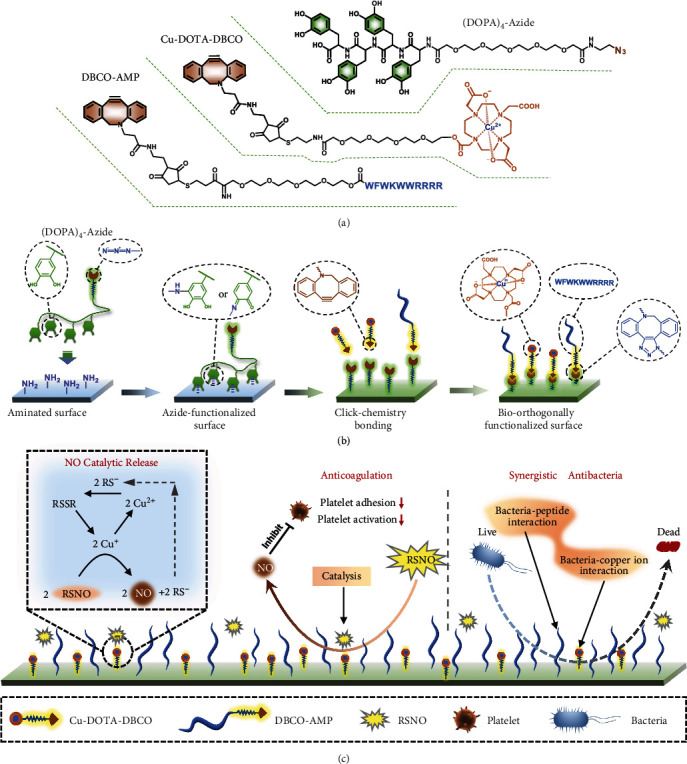
Fabrication of Cu-DOTA&AMP surface with anticoagulation and antibacterial properties. (a) Chemical structure of the clickable mussel-inspired peptide [(DOPA)_4_-azide, Ac-(DOPA)-Gly-(DOPA)-(Lys-PEG_5_-Azide)-(DOPA)-Gly-(DOPA)-COOH)], DBCO-modified antimicrobial peptide (DBCO-AMP), and DBCO-capped NO catalyst (Cu-DOTA-DBCO). (b) Surface cografting on representative medical devices through mussel-inspired catechol-amine reaction and bioorthogonal click chemistry. (c) Realization of anticoagulation and synergistic antibacterial properties of Cu-DOTA&AMP surface.

Herein, we combined mussel-inspired molecule and bio-orthogonal click chemistry for synergistically tailoring extracorporeal circuits and indwelling medical devices for antibacterial and anticoagulant multifunctions (Figure 1). Clickable mussel-inspired peptide Ac-(DOPA)-Gly-(DOPA)-(Lys-PEG_5_-Azide)-(DOPA)-Gly-(DOPA)-COOH (i.e., (DOPA)_4_-Azide) was chemisorbed onto a preaminated surface to impart the surface with sufficient azide groups for subsequently conjugating of DBCO-modified molecules (i.e., Cu-DOTA-DBCO and DBCO-AMP) by bioorthogonal N_3_-DBCO click reaction. The Cu-DOTA could catalytically generating NO by which the platelet adhesion/aggregation could be strongly inhibited. Moreover, the grafted AMP possesses high-efficiency capabilities of killing bacteria. Thus, the bio-orthogonally coimmobilization of Cu-DOTA and AMP is probably endowing the modified tubing with favored antimicrobial and antithrombotic dual functions. This design integrates NO-generating and antimicrobial peptide moieties into a tubing coating system by mussel-inspired adhesive peptide mimicking and bio-orthogonal click chemistry while involving only simple, specific, rapid, and reproducible procedures. Importantly, the Cu-DOTA-catalyzed NO generation and AMP might contribute synergistically and successfully to long-term antibacterial and antithrombosis. It is expected that this strategy can provide a facile approach for rational bioengineering of tubing and catheters with optimal multifunctions combating thrombosis and infection.

## 2. Results

### 2.1. Molecular Synthesis and Surface Functionalization

**Figure 2:**
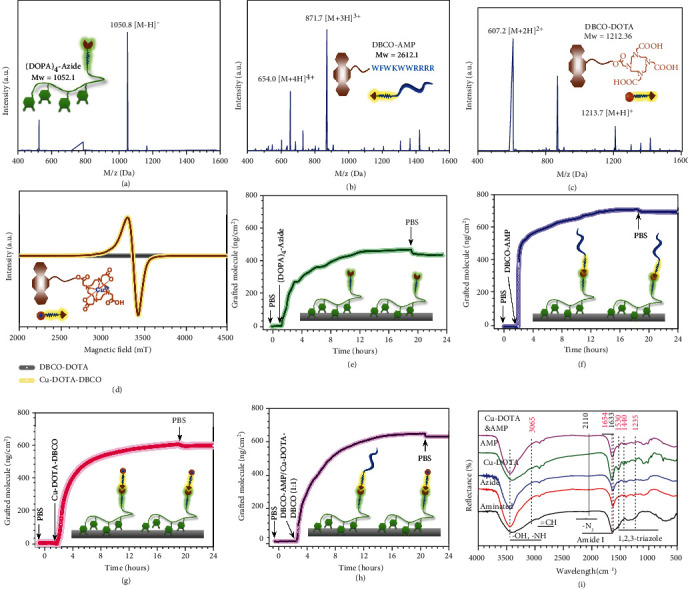
Synthesis and grafting of (DOPA)_4_-azide, DBCO-AMP, and Cu-DOTA-DBCO molecules on aminated surface. (a)–(c) Electrospray ionization mass spectrum of (DOPA)_4_-azide, DBCO-AMP, and DBCO-DOTA. (d) ESR spectrum of DBCO-DOTA with or without chelated copper ion. (e) Real-time monitoring of the grafting amount of (DOPA)_4_-azide on aminated surface using QCM. (f)–(h) Real-time monitoring of the grafting amount of DBCO-AMP, Cu-DOAT-DBCO, and DBCO-AMP/Cu-DOTA-DBCO on azide surface using QCM. (i) RA-FTIR spectra of aminated surface after grafting with (DOPA)_4_-azide, DBCO-AMP, Cu-DOAT-DBCO, and DBCO-AMP/Cu-DOTA-DBCO. Data are presented as mean ± SD (*n* = 4).

The clickable mussel-inspired peptide was prepared through standard Fmoc-mediated solid-phase peptide synthesis, as reported previously [33–36]. Briefly, to simulate the multiple catechol structure in Mfps [37, 38], acetonide-protected fluorenylmethyloxycarbonyl-DOPA(acetone)-OH was employed in introducing DOPA into the mussel-inspired peptide sequence. In order to promote the mussel-like molecular adhered to the substrates and leave available clickable groups for the following click reaction, tetravalent DOPA was integrated using an amino acid spacer and a PEG-connected azide to acquire a bioclickable mussel-inspired adhesive peptide (DOPA)_4_-azide. In this work, as two key active molecules for anticoagulation and antibiosis, the nitric oxide- (NO-) generating species Cu-DOTA and the AMP were connected to a PEG fragment with the DBCO group, respectively, to acquire the DBCO-capped biofunctional molecules. To obtain high purity of biofunctional molecules, the purity of (DOPA)_4_-azide, DBCO-AMP, and DBCO-DOTA were purified (99.06%, 96.02%, and 96.97%, respectively) by high-performance liquid chromatography (HPLC) (Figure S1). After purification through HPLC, the three chemical-synthesized molecules were then characterized with nuclear magnetic resonance (NMR) spectrometry and electrospray ionization mass spectrometry (ESI-MS). As expected, the monoisotopic mass [M-H]^−^ of (DOPA)_4_-azide, [M +3H]^3+^ of DBCO-AMP, and [M +2H]^2+^ of DBCO-DOTA was detected at 1050.8, 871.7, and 607.2 Da, meeting their theoretical molecular weight 1052.1, 2612.1, and 1212.36, respectively (Figures 2(a)–2(c)). ^1^H-NMR analysis also showed all diagnostic peaks of the three synthesized molecules and further confirmed the success of molecular synthesis (Figure S2). The results verified the successful chemical synthesis of the bioclickable adhesive peptide and DBCO-modified biofunctional molecules. To confirm the formation of the Cu-DOTA-DBCO molecule, the measurement of electron paramagnetic resonance (EPR) was carried out. EPR analysis revealed that Cu^2+^ was successfully chelated to the DBCO-DOTA, with the signals showing up at 3490-3430 mT (Figure 2(d)).

Currently, most of the indwelling medical devices and extracorporeal circuits used in clinical are made of polymer materials. However, there have limited surface bioengineering strategies for polymeric devices as compared with the metal devices, possibly owing to the chemical inertness of biomedical polymer materials. To facilitate the surface functionalization of polymer material, in this study, a durable amine-containing coating [39] was performed on the surface of polyvinyl chloride (PVC, a conventional medical materials approved for manufacturing blood-contacting device) substrates or tubes. Then, the mussel-inspired adhesive bioclickable peptide was robust tethered on the PVC through catechol-amine reaction. Finally, the azide-functionalized PVC surfaces were easily connected with the obtained DBCO-capped NO catalyst (Cu-DOTA-DBCO) and DBCO-modified antimicrobial peptide (DBCO-AMP) by DBCO-N_3_ bio-orthogonal reaction. The peptide binding and biomolecular grafting were monitored by Quartz Crystal Microbalance Dissipation Method (QCM-D). The results in Figure 2(e) demonstrated that 430.8 ng·cm^−2^ of (DOPA)_4_-azide was steadily bound onto the aminated chips, implying the high efficiency and robust tethering onto the aminated PVC substrates. Then, the azide-modified chips were incubated with DBCO-AMP or Cu-DOTA-DBCO via bio-orthogonal conjugation. Analysis of QCM-D monitoring revealed that the grafting processes began within minutes, and the maximal grafting amount for DBCO-AMP and Cu-DOTA-DBCO was 696.9 and 594.8 ng·cm^−2^, respectively (Figures 2(f) and 2(g)). Dual functionalization using a mixture of DBCO-AMP and Cu-DOTA-DBCO (1 : 1 in molar ratio) was further carried out, and the coimmobilized amount demonstrated a median value around 637.4 ng·cm^−2^ (Figure 2(h)).

To further investigate the changes of the chemical structure and composition of the aminated PVC before and after each grafting step, reflection absorbance Fourier transform infrared (RA-FTIR) and X-ray photoelectron spectroscopy (XPS) were carried out. After grafting of (DOPA)_4_-azide to the aminated surface (marked as azide), the introduction of −N = N^+^ = N^−^ peak by (DOPA)_4_-azide with azide groups in the FTIR spectra was nearly invisible, which may be due to the −N = N^+^ = N^−^ signal was limited by the detection limit of infrared. However, the band from 3100 to 3600 cm^−1^ was remarkably broadened because of the -OH stretching vibration bands derived from the (DOPA)_4_-azide containing a moderate amount of phenolic hydroxyl and carboxyl groups, probably indicating the successful grafting of (DOPA)_4_-azide. The click-anchoring sites for DBCO-modified biofunctional molecules were provided through introducing the azide groups to the aminated surface. After clicking of Cu-DOTA-DBCO or/and DBCO-AMP to azide (marked as Cu-DOTA, AMP, and Cu-DOTA&AMP, respectively), the appearance of the band in the FTIR spectra at 1530, 1440, and 1235 cm^−1^ confirm the 1,2,3-triazole group produced by the successful bioorthogonal N_3_-DBCO click reaction between the -N_3_ groups provided by azide coating and the -DBCO groups of Cu-DOTA-DBCO or DBCO-AMP. Additionally, the appearance of band at 3060 cm^−1^ (=CH stretching) and the significant shift of the peak from 1633 to 1654 cm^−1^ further indicated the successful bioconjugation of AMP. Although the band at 3060 cm^−1^ (=CH stretching) in the FTIR spectrum of Cu-DOTA&AMP nearly disappeared probably due to the successful clickable grafting of both molecules, the presence of similar significant shift of the band from 1633 to 1654 cm^−1^ also further confirmed the successful bioconjugation of Cu-DOTA-DBCO and DBCO-AMP on azide surface (Figure 2(i)). XPS analysis provided additional confirmation of the successful surface engineering of (DOPA)_4_-azide, DBCO-AMP, and Cu-DOTA-DBCO, as evidenced through the remarkable changes in the chemical compositions of the aminated PVC before and after each grafting step (Table S1). The changes in the contents of oxygen and nitrogen of the azide confirmed the immobilization of (DOPA)_4_-azide on the aminated coating. Moreover, the copper element especial to the Cu-DOTA-DBCO was tested in both the Cu-DOTA-containing coatings, and the significant decrease in the contents of the copper of the Cu-DOTA&AMP coatings compared with the Cu-DOTA coatings, which probably confirmed the effective bioconjugation of Cu-DOTA-DBCO and DBCO-AMP on the azide coating. To further investigate the chemical components of the functionalized surfaces, the C 1 s and N 1 s peaks of XPS were implemented with peak fitting methods (Figure S3). An increase of the peak was found at 286.2 eV in the C 1 s high-resolution spectra of the azide surface compared to that of aminated surface, which may be due to the introduction of C-O/C-N_3_ peak components by (DOPA)_4_-azide with azide groups and C-O structures. Additionally, an obvious decrease or increase of peaks value was found at 284.6 eV and 286.2 eV in the C 1 s high-resolution spectra of the AMP and Cu-DOTA surfaces compared to that of azide because of the introduction of C=C/C-C structure of the DBCO-AMP and Cu-DOTA-DBCO, implying the conjugation of AMP and Cu-DOTA on azide surfaces (Figure S3A). Finally, the content of each component in Cu-DOTA&AMP coatings was between the DOTA-Cu and AMP surfaces, which was consistent with the theoretical result of bioconjugation Cu-DOTA-DBCO and DBCO-AMP (Table S2). The N 1 s core-level spectrum of the aminated surface after grafting of (DOPA)_4_-azide was curve-fitted into five peak components with BEs at 398.07, 399.50, 400.46, 401.13, and 403.90 eV, attributable to the aromatic N, aliphatic N, Azide (N=N^−^), R-N^+^, and Azide (-N^+^) species, respectively (Figure S3B). The azide [(N) − N = ]/[−N^+^] area ratio of around 2 : 1 was in good agreement with the characteristics of azide [40], indicating that the mussel-inspired adhesive bioclickable peptide had grafted on the aminated surface. Moreover, the absorption of nonazide protonated R-N^+^ was significantly enhanced, which probably is due to the aniline structure formed by Michael addition between the -NH_2_ groups of aminated surface and the benzodiazepines groups of (DOPA)_4_-azide was easily protonated. Therefore, the disappearance of azide (-N^+^) and ((N)-N=) species of the AMP, Cu-DOTA, and Cu-DOTA&AMP surfaces further indicated that the Cu-DOTA-DBCO and DBCO-AMP were successfully clicked on the azide-functionalized surface via bioorthogonal N_3_-DBCO click reaction (Table S3).

Altogether, in the above results, the potential of bio-orthogonal conjugation biofunctional molecule for fabrication of dual-functional surface was verified.

### 2.2. Antibacterial Property

**Figure 3:**
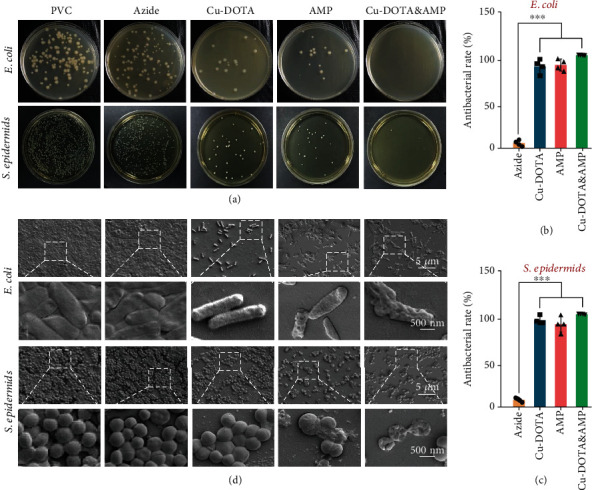
Antibacterial property. (a) Typical E. coli and S. epidermids colonies after 24 h incubation on the bare and modified PVC plates. Antibacterial rate of modified PVC that calculated from the number of colonies against (b) E. coli and (c) S. epidermids. (d) SEM images of E. coli and S. epidermids that adhered or colonized on the bare and modified PVC plates. All scale bars from the same raw are 500 or 5000 nm. Data presented as mean ± SD and analyzed using a one-way ANOVA, ∗∗*p* < 0.01, ∗∗∗*p* < 0.001.

Infection is one of the major complications associated with extracorporeal circuits and indwelling medical devices, which causes significant mortality in clinic [41]. To detect the antibacterial performance of the Cu-DOTA&AMP coating, Escherichia coli (E. coli) and Staphylococci epidermids (S. epidermids) were selected as typical strains causing infections after interventional procedures for antibacterial tests. We found that the surface azidation to the PVC substrates by (DOPA)_4_-azide did not result in visible influence on bacterial growth as evidenced by antibacterial rates of 9.2% and 9.0% for E. coli and S. aureus, respectively. However, the Cu-DOTA-grafted on azide-functionalized PVC led to inhibitory effects on bacterial growth, which may be attributed to the bactericidal ability of the leakage copper(II)-ions from Cu-DOTA. Moreover, a significantly growth of the antibacterial rate in AMP-coated PVC compared to the control and azide groups was also observed. In addition, we have noted that the bio-orthogonal of Cu-DOTA&AMP on azide-functionalized PVC proved the excellent synergistic interactions on inhibiting S. epidermids and E. coli with bacterial killing activity nearly to 99% and 100%, respectively (Figures 3(a)–3(c)).

SEM analysis showed that a moderate number of S. epidermids and E. coli adhered on both PVC and azide groups showed a state of rapid proliferation (Figure 3(d)). In contrast, the surfaces functionalized by Cu-DOTA or AMP alone significantly inhibited the adhesion and proliferation of S. epidermids and E. coli. We have not noted that the bacteria membrane ruptured obviously on the Cu-DOTA, which might be owing to the insufficient concentration of free of copper ions from Cu-DOTA. However, it was noteworthy that the bacterial membrane rupture was observed on the AMP and Cu-DOTA&AMP, which demonstrated the well-retained ability of AMP [42] grafted by surface click chemistry to penetrate and destroy the bacterial membrane for killing bacteria. In addition, the excellent synergistic effects on the adhesion and proliferation of S. epidermids and E. coli were exhibited at the bio-orthogonal of Cu-DOTA&AMP on azide-functionalized PVC surface.

Considering the long-term practical applications of the Cu-DOTA&AMP coating on implantable medical blood-contacting devices (e.g., central venous catheter and pacemaker), the long-term efficacy of its antibacterial efficacy for different time period at continuous immersion into PBS was tested (Figure S4). The results revealed that antibacterial rates of Cu-DOTA&AMP-coated PVC for *S. epidermids* and *E. coli* after 30 days of soaking, but still suppressed 98% of bacterial growth of both S. epidermids and E. coli, implying the wonderful preservation of Cu-DOTA&AMP in antibacterial properties.

Altogether, the results above shows that the AMP and Cu^2+^ from Cu-DOTA endow the long-term antibacterial properties of Cu-DOTA&AMP surface to prevent biofouling.

### 2.3. *In Vitro* NO Catalytic Release and Blood Compatibility Tests

**Figure 4:**
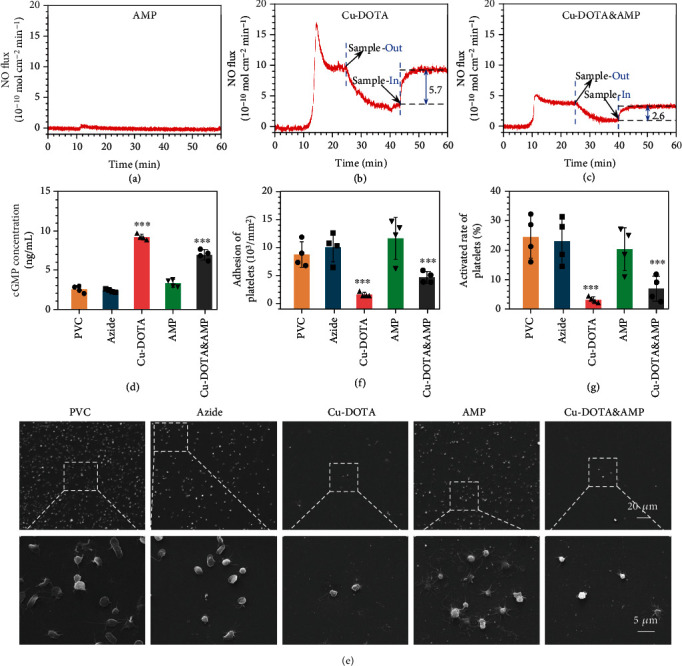
In vitro NO catalytic release and In vitro blood compatibility tests. NO generation rate of (a) AMP, (b) Cu-DOTA, and (c) Cu-DOTA&AMP monitored by real-time NOA. (d) Concentration of cGMP synthesized by platelets. (e) SEM images (all scale bars from the same raw are 20 or 5 *μ*m), (f) adhesion number, and (g) activation rate of the adhered platelets on bare and modified PVC. Data presented as mean ± SD and analyzed using a one-way ANOVA, ∗∗*p* < 0.01, ∗∗∗*p* < 0.001.

In our design, the DOTA chelated copper ions can decompose nitrosothiols into NO to suppress material-induced thrombosis [25, 26, 43]. The catalytic release of NO as bioactive gas molecule was calculated to detect the NO catalytic ability of the Cu-DOTA&AMP coating. The NO catalytic release activity of Cu-DOTA&AMP surface was evaluated through a real-time chemiluminescent assay. For the in vitro experiments, the NO donor solution (10 *μΜ* S-nitrosoglutathione (GSNO) and 10 *μΜ* L-glutathione (GSH)) was prepared to mimic the physiological environment. NO real-time monitoring revealed that there is almost no catalytic release of nitric oxide from the AMP-coated PVC (Figure 4(a)). As shown in Figures 4(b) and 4(c), the stable NO release patterns yield by the Cu-DOTA-coated PVC (5.7 × 10^−10^ mol cm^−2^ min^−1^), suggesting the successful functionalization of in situ release of NO. In contrast, the Cu-DOTA&AMP surface showed slightly lower NO flux (2.6 × 10^−10^ mol · cm^−2^ · min^−1^) possibly due to the successful clickable grafting of both molecules. Considering the stable catalytic release of NO is critical to the implanted blood-contacting devices for long-term service *in vivo*, stability studies were prepared for Cu-DOTA&AMP through immersion into PBS for different time periods, and then their NO catalytic release ability was tested every seven days. The experimental result indicates that the NO catalytic release ability exhibited a minor decrease with the increase of soaking time, but stabilized around (0.5 ~ 1) × 10^−10^ mol · cm^−2^ · min^−1^ after more than 30 days (Figure S5). Jointly, these results confirmed the possibility of the dual-functional Cu-DOTA&AMP surfaces for long-term service *in vivo*.

At the early postimplantation stage, thrombosis is a crucial problem associated with blood-contacting material. As a highly potent gaseous signal molecule, NO can activates soluble guanylate cyclase through binding the haem moiety of soluble guanylate cyclase, contributing to cyclic guanosine monophosphate (cGMP) upregulation and eventually inhibiting the activation and aggregation of platelets [44]. To evaluate the ability of NO produced from Cu-DOTA on facilitating the cGMP synthesis of platelets, the cGMP analysis was carried out. Compared to the PVC and azide groups, only the groups (Cu-DOTA and Cu-DOTA&AMP) containing Cu-DOTA both significantly facilitated the cGMP expression of platelets with the NO donor supplement, and the grafted only AMP group had no charge (Figure 4(d)). Moreover, we also noted a slight loss in the cGMP expression due to the loss of NO released by Cu-DOTA&AMP (Figure 4(c)). In contrast, the expression of cGMP of platelets did not change without NO donor supplement (Figure S6A), indicating the highly biological activity of NO generated from Cu-DOTA. Then, we further investigated the adhesion and activation of platelets on the dual-functional Cu-DOTA&AMP surface. Without donor supplement, a moderate number of activated platelets aggregate on all the experiment groups (Figure S6B-D). Upon adding donor to catalytic release NO, most of platelet adhered on both PVC and azide groups with a state of remarkable degree of activation and aggregation was observed. Furthermore, the grafted AMP-only group still had obvious platelet adhesion and activation. By contrast, the grafted Cu-DOTA group substantially reduced platelet adhesion and activation with an inactive spherical state. Though the generation of NO induced by Cu-DOTA&AMP was a slight decrease compared with the grafted Cu-DOTA group, it still remarkably inhibited the adhesion and activation of platelet (Figures 4(e)–4(g)). the result suggested the efficacy of NO gas molecules for the suppression of thrombogenesis in blood microenvironment.

Altogether, these results demonstrate the excellent antithrombogenic properties of the Cu-DOTA&AMP surface in vitro.

### 2.4. *Ex Vivo* Antithrombogenic Properties

**Figure 5:**
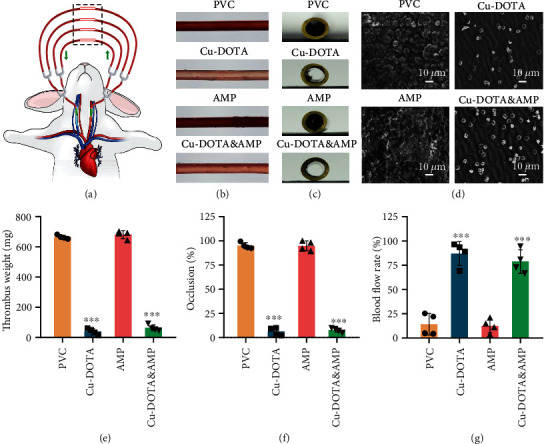
Ex vivo hemocompatibility of the Cu-DOTA&AMP surfaces. (a) Schematic illustration of arteriovenous (AV) shunt model connected to the rabbit. Photographs of (b) side and (c) cross-section view of the bare and modified PVC tubes after blood circulation. (d) Lumen surface morphology of each sample after blood circulation characterized by SEM. (e) Thrombus weight, (f) occlusion, and (g) blood flow rate of bare and modified PVC tubes following the blood circulation. Data presented as mean ± SD and analyzed using a one-way ANOVA, ∗∗*p* < 0.01, ∗∗∗*p* < 0.001.

To be more clinically relevant, an *ex vivo* blood circuit experiment was further performed [45]. The PVC tubing before and after surface engineering was assembled with clinically usable medical catheter and subsequently linked to a rabbit arteriovenous shunt circuit (Figure 5(a)).

With the NO donor supplement, all the tubings were collected to evaluate the occlusive rates, blood flow rates, and thrombus weights after ex vivo circulation for 2 h. We noted that there was only a tiny number of thrombi on the groups with the NO releasing, whereas severe thrombus formation was observed on the NO-free groups (PVC and AMP) (Figures 5(b) and 5(c)). SEM analysis further confirmed that the groups containing Cu-DOTA significantly prevented the formation of thrombus. On the blood-contact surfaces of the bare PVC tubing and AMP-coated tubing, there had severe thrombus with high crosslinking density of polymeric fibrin networks, red blood cells, and activated platelets (Figure 5(d)). Quantitative of these results further revealed a significant reduction in thrombosis formation of the NO releasing groups through evaluating thrombus weight, occlusion rates, and blood flow rates relative to the other groups (Figures 5(e)–5(g)). Total thrombus weight in NO releasing circuits was ten-fold reduced compared to the bare and only AMP-grafted groups (Figure 5(e)). To conclude the occlusion rates throughout the circuits postexplant, the percent occlusion of lumen areas was calculated using computerized image analysis. Compared to the bare and only AMP-grafted groups, the Cu-DOTA&AMP coated circuit significantly reduced the thrombotic occlusion to 10% (Figure 5(f)). Correspondingly, the blood flow rates revealed a consistent result (Figure 5(g)). These results were also in line with the antithrombogenic property in vitro, described above.

Considering the long-term practical applications of the Cu-DOTA&AMP coating on implantable medical blood-contacting devices (e.g., pacemaker and central venous catheter), the long-term efficacy of its anticoagulant efficacy for different time periods at continuous immersion into PBS was tested (Figure S7). The results showed that the antithrombogenic ability showed an almost no decrease after 30 days of treatment with PBS, suggesting the promising application of Cu-DOTA&AMP coating in implanted/interventional blood-contacting devices for long-term use in vivo.

The above results also confirmed the excellent antithrombogenic property of the Cu-DOTA&AMP surface long-term use in vivo.

### 2.5. Blood Biochemical Analysis

**Figure 6:**
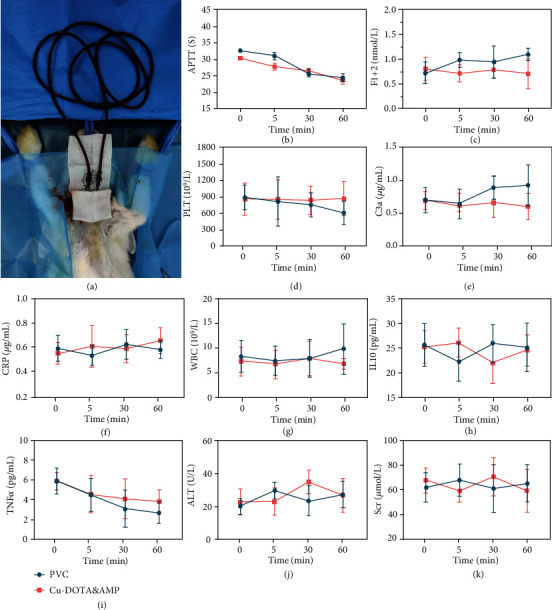
Blood analysis by ex vivo blood circulation. (a) Circulation model on rabbit for blood analysis. Blood parameters including (b) activated partial thromboplastin time (APTT), (c) *F*1 + 2 (an integral marker for the prothrombin activation), (d) the number of platelets (PLT), (e) C3a (C3 cleavage fragment, indicating the classic or alternative way to activate the complement system), (f) c-reactive protein (CRP), (g) white blood cells (WBC), (h) IL-10 (a recognized inflammatory and immunosuppressor), (i) tumor necrosis factor-alpha (TNF-*α*), (j) the liver enzyme alanine aminotransferase (ALT), and (k) the kidney parameter serum creatinine (Scr) were detected to reveal the hemocompatibility and liver/kidney safety after exposing the bare and Cu-DOTA&AMP grafted catheters to the circulating blood. Data are presented as mean ± SD (*n* = 4).

Antithrombosis is the major property associated with blood-contacting materials in addition to the hemostatic materials. However, for those biomedical devices which undergo long-term or large-area contact with blood, e.g., the central venous catheter or cardiac pacemaker, their effects on blood composition or on liver and kidney functions should be systematically detected to ensure the safety for use in vivo. Considering the difference of total blood volume between rabbit and human (~200 ml for mature white rabbit and ~4000 ml for adult human), a 1.6 m long bare PVC or Cu-DOTA&AMP-coated tube (with inner diameter 3 mm) was selected to simulate the clinical application, and then, respectively, installed into the rabbit arteriovenous shunt circuit (Figure 6(a)). After 0, 5, 30, and 60 min of blood circulation, the blood was collected for biochemical, and physiological parameters were determined, including inflammatory response, blood coagulation, and organ functions.

Coagulation evaluation showed that the control groups had a trend towards a higher blood clotting after prolonged contact with blood, which was characterized by the increased amount of F1+2 (an integral marker for the prothrombin activation) (Figure 6(c)). However, activated partial thromboplastin time (APTT) decreased significantly in all groups, probably because that the catalytic release of NO was insufficient (Figure 6(b)). After circulation, there had been a decrease in the number of platelets of bare PVC tubing, but the number of platelets of the Cu-DOTA&AMP-coated PVC tubing did not change (Figure 6(d)). A device implanted in vivo can be quickly monitored by the immune system, then causing an inflammatory response. While the proinflammatory parameters of all groups had no obvious changes: the count of C3a (C3 cleavage fragment, indicating the classic or alternative way to activate the complement system, Figure 6(e)), c-reactive protein (a kind of acute-phase proteins in plasma were used as a measurement of acute inflammation, Figure 6(f)), white blood cells (Figure 6(g)), expression of tumor necrosis factor-alpha (TNF-*α*) (a major acute inflammatory cytokines, Figure 6(i)), and IL-10 (a recognized inflammatory and immunosuppressor, Figure 6(h)). Such results indicate that all groups had no proinflammatory tendency. However, only the expression of TNF-*α* decreased significantly after cycling, which may be related to the anesthesia [46]. All the proinflammatory indexes of the Cu-DOTA&AMP surface group had a consistent result with the bare PVC group used in clinical, suggesting that the coating did not further promote an inflammatory response of the material. To explore if the material and coating had serious toxicity to organs and tissues, the blood concentrations of the liver enzyme alanine aminotransferase (ALT) and the kidney parameter serum creatinine (Scr) were measured. As shown in Figures 6(j) and 6(k), both the PVC and Cu-DOTA&AMP surface presented no organ and tissue toxicity during circulation, and there was also no significant difference, verifying their biosafety.

## 3. Discussion

We develop here a biomimetic surface engineering strategy for fabricating multifunctional coating onto blood-contacting surfaces by masterly combining bioorthogonal conjugation chemistry with mussel-inspired adhesive peptide mimicking. To robustly binding clickable mussel-inspired peptide on tubing and catheters surface by catechol-amine chemistry, we functioned the polymeric device with a durable amine-bearing surface. The DBCO-modified functional molecules (e.g., Cu-DOTA-DBCO and DBCO-AMP) were effectively coimmobilized on tubing and catheter surface through the bio-orthogonal conjugation chemistry. The antibacterial function of DBCO-AMP and the signal molecules effect of NO generated from Cu-DOTA-DBCO endowed the coating with the durable synergistic inhibition in growth and adhesion of bacteria and activation of platelets in vitro for one month, as well the capacity to efficiently restrain thrombogenesis *ex vivo*. Our strategy presented a promising method to tailor a multifunctional surface and maintain efficient biological function and may also be suitable for biomimetic surface engineering of many biomedical fields.

## 4. Materials and Methods

### 4.1. Materials

Dopamine, polyallylamine, CuCl_2_·2H_2_O, GSNO, GSH, cGMP enzyme immunoassay kit, and glutaraldehyde were purchased from Sigma-Aldrich.

### 4.2. Surface Amination of PVC

Briefly, the PVC substrates (1.0 cm × 1.0 cm for antiplatelet test and 2.5 cm × 2.5 cm for antibacterial assessment) or tubes (inner diameter *Φ* = 3.0 *mm*) were dipped into or perfused with the dopamine (DA) solution under optimal conditions (10 mM Tris-HCl, pH 8.5, 1 mg mL^−1^ DA) for 48 h. Then, the polydopamine- (PDA-) modified PVC substrates or tubes were flushed with deionized water and desiccated using N_2_ gas. To further tailor surface amino functionalization of the PVC, the modified PVC substrates or tubes were subsequently soaked into the polyallylamine alkaline aqueous solution (pH = 12) under ambient temperature for 12 h and finally cleaned with distilled water and desiccated by N_2_ for future use.

(DOPA)_4_-Azide, DBCO-AMP, and DBCO-DOTA Synthesis: the (DOPA)_4_-azide and the DBCO-capped molecules (DBCO-AMP and DBCO-DOTA) were prepared through the Fmoc-mediated solid-phase synthesis technique [47]. With the help of China Peptides Co. Ltd. (Shanghai, China, purity > 95%). The Cu-DOTA-DBCO was prepared by mixing the CuCl_2_·2H_2_O with DBCO-DOTA in the aqueous solution by a mol ratio of 1 : 1.

### 4.3. Surface Azidation of the PVC and Cografting of the Cu-DOTA-DBCO and DBCO-AMP

The aminated PVC substrates or tubes (termed as aminate) were firstly immersed into DOPA_4_-Azide (0.1 mg mL^−1^) dissolved by PBS (pH = 8.0) under ambient temperature for 24 h, and then cleaned by deionized water and desiccated with a stream of N_2_, the azide-modified surface was termed as “Azide.” Subsequently, the azide-modified PVC was incubated with 2 mg mL^−1^ of Cu-DOTA-DBCO and/or 2 mg mL^−1^ DBCO-AMP under ambient temperature for 24 h to prepare the Cu-DOTA, AMP, and Cu-DOTA&AMP cografted surface. After cografting, the modified substrates or tubes were cleaned thorough deionized water and desiccated with a stream of N_2_ for subsequent use.

### 4.4. Characterization

To purify the synthesized molecules, HPLC was performed on an Agilent HPLC system with a Kromasil 100-5C_18_ column (5 *μ*m, 4.6 × 250 mm, column temperature 25°C). Mobile phases were buffer A (0.1% TFA in water) and buffer B (0.1% TFA in acetonitrile). The flow was graded at a rate of 1 mL min^−1^. Then, injection volume and running time were 10 *μ*L and 11 min, respectively. The molecular weights of (DOPA)_4_-azide, DBCO-AMP, and DBCO-DOTA were determined by ESI-MS (Sciex API 150EX LC/MS with Agilent 1100 HPLC). Buffer: 75%ACN/24.5%H_2_O/0.5%Ac; flow rate: 0.2 mL min^−1^; run time: 1 min. A Nicolet model 5700 instrument was used to take Reflection Absorbance Fourier Transform Infrared (RA-FTIR) spectrum. The surface elemental compositions were detected by X-ray photoelectron spectroscopy (XPS) (K-alpha, Thermo Fisher, USA), with an excitation source monochromatic Al K*α* (1486.6 eV). The synthesized molecules were analyzed by proton nuclear magnetic resonance (^1^H-NMR) spectrum (Bruker AVANCE III 400). 10 mg sample was used to measure electron paramagnetic resonance (EPR) spectra on a Bruker EPR EMXPlus (X-band is 9.85 GHz, field modulation is 100 kHz, the power is 0.2 mW, and samples were loaded in capillary tube).

### 4.5. QCM-D Analysis

The real-time monitoring of the grafting of the (DOPA)_4_-azide and the DBCO-capped molecules (DBCO-AMP or/and Cu-DOTA-DBCO) was used by a Quartz Crystal Microbalance Dissipation Method (QCM-D, Q-sense AB, Sweden). In detail, the Au-coated quartz crystal (diameter of Au film 1 cm) was firstly surface aminated. Then, the piezoelectric quartz crystal sensors were excited at a fundamental frequency (5 MHz), and the change in frequency (Δ*f*) was monitored for the third, fifth, seventh, ninth, eleventh, and thirteenth overtones. When the excitation stopped, recorded the changes of the resonance frequencies (Δ*F*) and those of the relaxation (Δ*D*) of the vibration at the five frequencies, and installed the surface aminated quartz crystal in the QCM-D chamber and injected PBS buffer at 50 *μ*L min^−1^ continuously until the QCM-D traces maintained steady, followed by the buffer pump with the identical speed. After that, (DOPA)_4_-azide (0.1 mg mL^−1^) in PBS was injected into the surveying chamber in touch with the crystal with the identical speed and eventually washed with PBS. Subsequently, 2 mg mL^−1^ of Cu-DOTA-DBCO and/or 2 mg mL^−1^ of DBCO-AMP were injected into the surveying chamber in touch with the crystal with the identical speed and eventually washed with PBS. Finally, the amounts of the Cu-DOTA-DBCO or/and the DBCO-AMP grafted on the surfaces were calculated based on the Sauerbrey equation [48].

### 4.6. Antibacterial Activity

The detailed procedure has been described elsewhere [49]. Briefly, *S. aureus* and *E. coli* were precultured in agar solid medium positioned in a 37°C incubator for 24 h and passaged twice to acquire the monoclonal bacterial. Picked the fresh bacterial colonies (1-3 rings) and dissolved with a solution containing 0.2% liquid medium and 99.8% saline. The bacteria concentration was restructured to 5.0 × 10^5^ ~ 10^6^ CFU mL^−1^ by tenfold increasing sequential diluting. 100 *μ*L of the suitable concentration of bacterial solution was added onto the surface of samples and covered using a soft polyethylene (PE) membrane to maintain a well spread liquid film on the substrate. All samples were positioned in a 37°C incubator for 24 h. Then, the bacteria on the surfaces were flushed and diluted with saline solution (1 mL). 20 *μ*L of the bacterial solution was distributed on the solid medium and incubated in a 37°C incubator. Lastly, the colonies cultured on the surface of solid medium were quantified and observed by SEM after 24 h.

### 4.7. Catalytic Generation of NO

A chemiluminescence NO analyzer (NOA, Seivers 280i, Boulder, CO) was used to determine the real-time generation rate of NO. In brief, the AMP, Cu-DOTA, and Cu-DOTA&AMP-modified PVC substrates (5 mm × 10 mm) were immersed into PBS with NO donor containing SNAP (10 *μ*M) and GSH (10 *μ*M). Upon the reaction was carried out, the NO-generated was conveyed into the NO analyzer by a stream of N_2_. Finally, the NO flux was calculated using the calibration line, which has been reported in details elsewhere [50].

### 4.8. Platelet Adhesion and Activation

The bare and AMP-, Cu-DOTA, and Cu-DOTA&AMP-modified PVC were cultured by 0.5 mL of platelet-rich plasma (PRP). After culture for 30 min at 37°C, the samples were rinsed 3 times with saline solution and then fixed with 4% paraformaldehyde solution overnight. After further dehydrated and dealcoholized, the adhered platelets on the sample surfaces were evaluated using scanning electron microscope (SEM) (ZEISS EVO 18). Given the low synthesis of endogenous in PRP, the samples were cultured in two groups of PRP supplemented with or without NO donor containing SNAP (10 *μ*M) and GSH (10 *μ*M).

### 4.9. cGMP Expression of Platelets

Expression level of cGMP of platelets adhered on each sample was measured by the human cGMP ELISA kit. Samples were firstly cultured in 1 mL of PRP at 37°C for 30 min with or without NO donor. Therewith, 100 *μ*L of triton-X solution (10%) was added onto the above sample and followed by sonication. The obtained above fragmentized PRP solution was centrifugated for 15 min at 3000 rcf, and the supernatant was collected for ELISA test.

### 4.10. *Ex Vivo* Hemocompatibility Test

The animal experiments obey the rules and guidelines of the China Ethical Committee and Laboratory Animal Administration Methods. The detailed experimental process has been described elsewhere [50]. Briefly, the Cu-DOTA-DBCO or/and DBCO-AMP were firstly immobilized on the lumen surface of the PVC tubings. After being anesthetized by 30 mg mL^−1^ pentobarbital sodium (1 mL per kg), the left external jugular vein and right carotid artery of New Zealand white rabbits (above 2.5 kg) were exposed and cannulated. Then, the catheter was linked to the cannulas, forming a closed loop. The unmodified-, and AMP-, Cu-DOTA, and Cu-DOTA&AMP-modified PVC tubings were taken out after *ex vivo* circulation for two hours and washed with saline solution. The occlusive rates were calculated by the cross-sections photographs of the tubings. Under the same pressure pump condition, the blood flow rate was calculated after circulation and normalized to that before the circulation. The thrombus formed on the luminal surface of the tubes was weighed, then analyzed by SEM after being fixed with 4% paraformaldehyde solution.

### 4.11. Blood Analysis after Ex Vivo Blood Circulation

The blood from New Zealand white rabbits was extracted for haematology analysis and blood biochemistry assay after ex vivo blood circulation. In the case of this experiment, each rabbit only received one circuit (e.g., unmodified PVC or Cu-DOTA&AMP-modified tubing). Blood analysis including the APTT [51], *F*1 + 2 [52], C3a [53], CRP [54], WBC, PLT, IL-10 [55], TNF-*α* [56], ALT [57], and CRE [58] was performed by using the blood drawn from the running circulation at different periods of time (0, 5, 30, and 60 min). A portion of the freshly collected whole blood with and without anticoagulants was centrifuged (2500 rcf at 4°C for 15 min) to collect plasma and serum, respectively. To test whether the coated catheter would have side effects on blood composition or on liver and kidney functions, a single prolonged catheter was selected to simulate the clinical application. The total ECC tube for human is several meters long, the area of blood-material interface of it is as high as10^2^-10^3^ cm^2^ as speculated. Considering the difference of total blood volume between rabbit and human (~200 ml for mature white rabbit and ~4000 ml for adult human), we selected a 1.6 meters long PVC tube with inner diameter = 3 mm and coated the lumen surface. The enlarged contact area facilitates us to disclose the different effect between the modified and unmodified (control) catheter.

APTT was measured by manual tilt-tube method [59]. The CRP, TNF-*α*, C3a, IL-10, and *F*1 + 2 were measured by ELISA kit (Rabbit CRP/TNF-*α*/C3a/IL-10/F1+2 ELISA KIT, ZC-52314/ZC-52984/ZC-52409/ZC-52381/ZC-52601, Shanghai ZCIBIO Technology Co., Ltd.), according to the specifications. WBC and PLT were measured by animal automatic blood cell analyzer (Shenzhen Mindray, BC-2800Vet). ALT and Scr were measured by animal biochemical analyzer (Shenzhen Mindray, BS-240VET).

### 4.12. Statistical Analysis

All the group data are expressed as mean with standard deviation for every sample unless specified otherwise. All the experiments were repeated independently at least three times, if not otherwise indicated. Student's *t*-test and one-way ANOVA in GraphPad Prism 9.0 (GraphPad Software) were performed for statistical analyses from different groups. Significance was denoted as follows: (ns: *p* > 0.05, ∗: *p* ≤ 0.05, ∗∗: *p* ≤ 0.01, ∗∗∗: *p* ≤ 0.001, ∗∗∗∗).

## Data Availability

All data needed to evaluate the conclusions in this paper are present in the paper and/or the Supplementary Materials. Additional data or materials related to this paper are available when requested.

## Supplementary Materials

Supplementary Materials Figure S1: high-performance liquid chromatography spectrum of (DOPA)4-azide, DBCO-AMP, and DBCO-DOTA. Figure S2: 1H NMR spectrum of (DOPA)4-azide, DBCO-AMP, and DBCO-DOTA. Figure S3: high-resolution spectra of C1s and N1s signals on the different surfaces. Figure S4: stability of the antibacterial ability of Cu-DOTA&AMP coating. Figure S5: stability of the NO catalytic release ability of Cu-DOTA&AMP coating. Figure S6: in vitro blood compatibility tests without NO donor. Figure S7: stability of the anticoagulation ability of Cu-DOTA&AMP coating. Table S1: the atomic compositions of different surfaces. Table S2: the high-resolution C1s compositions of different surfaces. Table S3: high-resolution N1s compositions of different surfaces.
