# Supplementary material for: Mussel-Inspired and Bioclickable Peptide Engineered Surface to Combat Thrombosis and Infection
Source: Research (Wash D C). 2022 Apr 14;2022:9780879. doi: 10.34133/2022/9780879 (PMC9034468; doi:10.34133/2022/9780879)
Supplement: Supplementary Materials — Figure S1: high-performance liquid chromatography spectrum of (DOPA)4-azide, DBCO-AMP, and DBCO-DOTA. Figure S2: 1H NMR spectrum of (DOPA)4-azide, DBCO-AMP, and DBCO-DOTA. Figure S3: high-resolution spectra of C1s and N1s signals on the different surfaces. Figure S4: stability of the antibacterial ability of Cu-DOTA& coating. Figure S5: stability of the NO catalytic release ability of Cu-DOTA& coating. Figure S6: in vitro blood compatibility tests without NO donor. Figure S7: stability of the anticoagulation ability of Cu-DOTA& coating. Table S1: the atomic compositions of different surfaces. Table S2: the high-resolution C1s compositions of different surfaces. Table S3: high-resolution N1s compositions of different surfaces. [file 9780879.f1.docx]

Mussel-Inspired and Bioclickable Peptide Engineered Surface to Combat Thrombosis and Infection

Xiaohui Mou^1,2,3^†, Hongbo Zhang^4^†, Hua Qiu^3^†, Wentai Zhang^1,2^, Ying Wang^1,2^, Kaiqin Xiong^3,5^, Nan Huang^3^*, Hélder A. Santos^6,7^*, and Zhilu Yang^1,2,3*^

*Corresponding author. Email: [zhiluyang1029@126.com](mailto:zhiluyang1029@126.com) (Z.L.Y); [huangnan1956@163.com](mailto:huangnan1956@163.com) (N.H); [h.a.santos@umcg.nl](mailto:h.a.santos@umcg.nl) (H.A.S)

**This PDF file includes:**

Supplementary Text

Figs. S1 to S7

Tables S1 to S3

Supplementary Text

Figure. S1. High-performance liquid chromatography spectrum of (DOPA)4-Azide, DBCO-AMP and DBCO-DOTA.

Figure. S2. 1H NMR spectrum of (DOPA)4-Azide, DBCO-AMP and DBCO-DOTA.

Figure. S3. High resolution spectra of C1s and N1s signals on the different surfaces.

Figure. S4. Stability of the antibacterial ability of Cu-DOTA&AMP coating.

Figure. S5. Stability of the NO catalytic release ability of Cu-DOTA&AMP coating.

Figure. S6. In vitro blood compatibility tests without NO donor.

Figure. S7. Stability of the Anticoagulation ability of Cu-DOTA&AMP coating.

Table S1. The atomic compositions of different surfaces.

Table S2. The high-resolution C1s compositions of different surfaces.

Table S3. High-resolution N1s compositions of different surfaces.


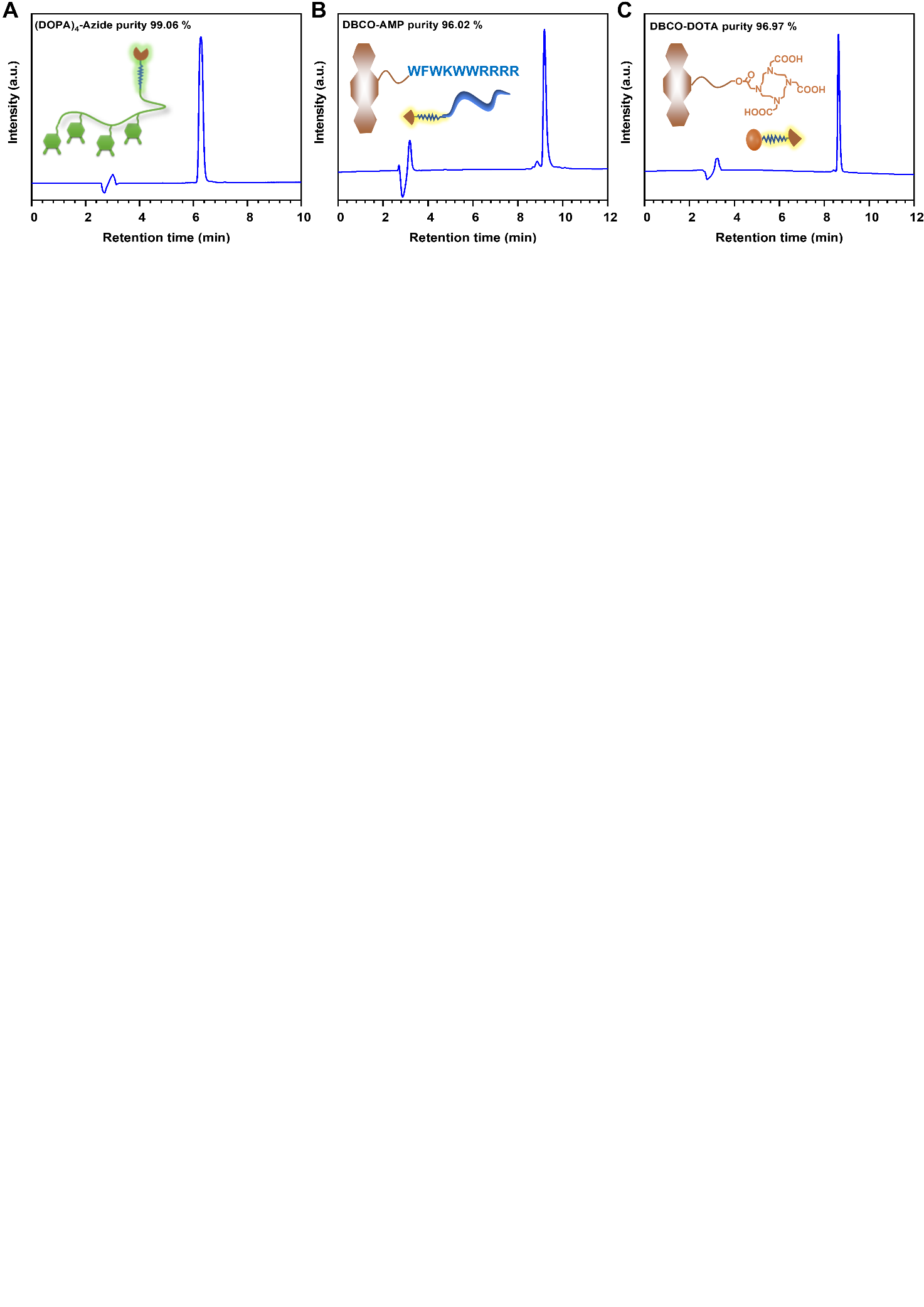


**Figure. S1. High-performance liquid chromatography spectrum** of (**A**) (DOPA)_4_-Azide, (**B**) DBCO-AMP and (**C**) DBCO-DOTA.


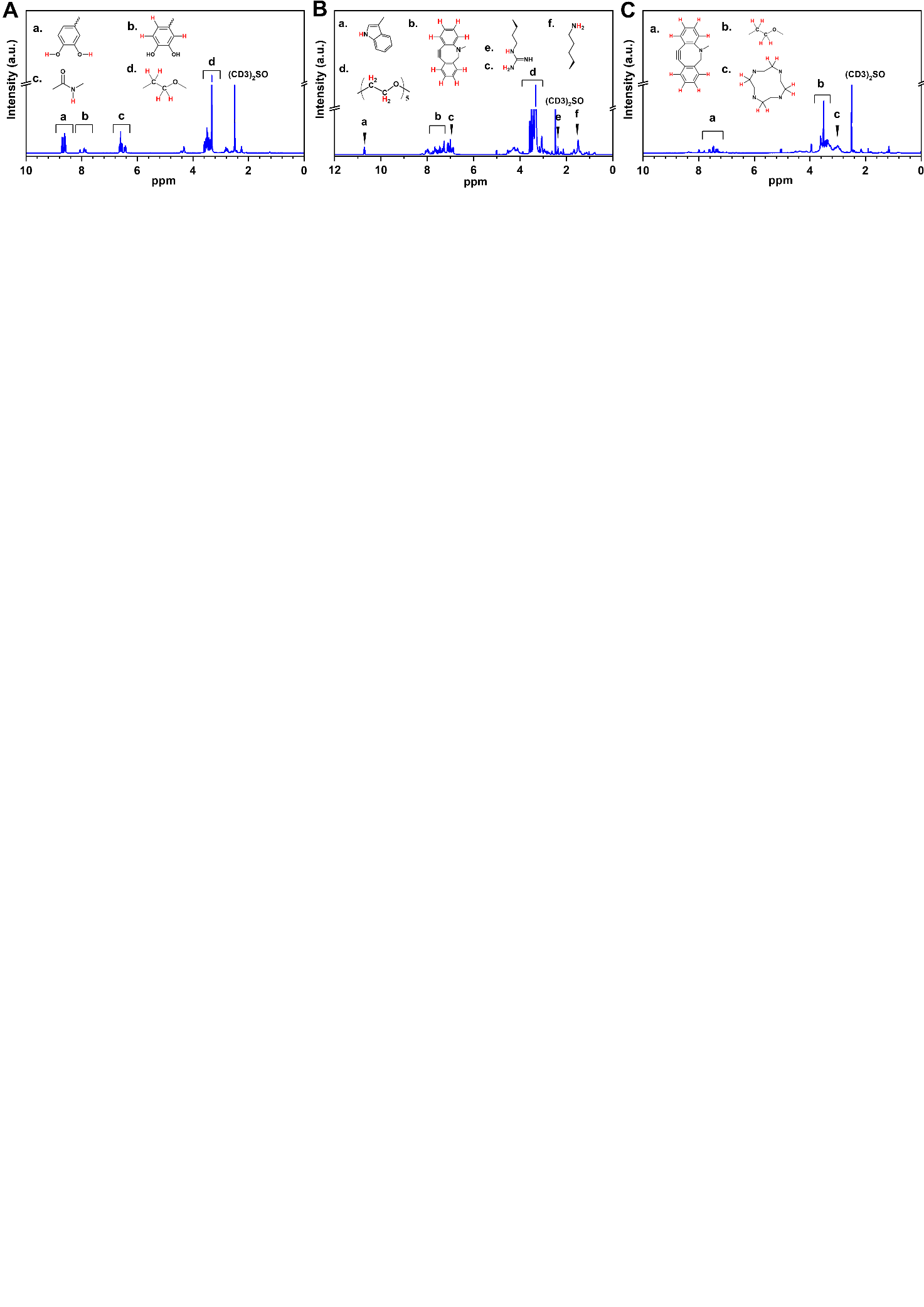


**Figure. S2. ^1^H NMR spectrum** of (**A**) (DOPA)_4_-Azide, (**B**) DBCO-AMP and (**C**) DBCO-DOTA.


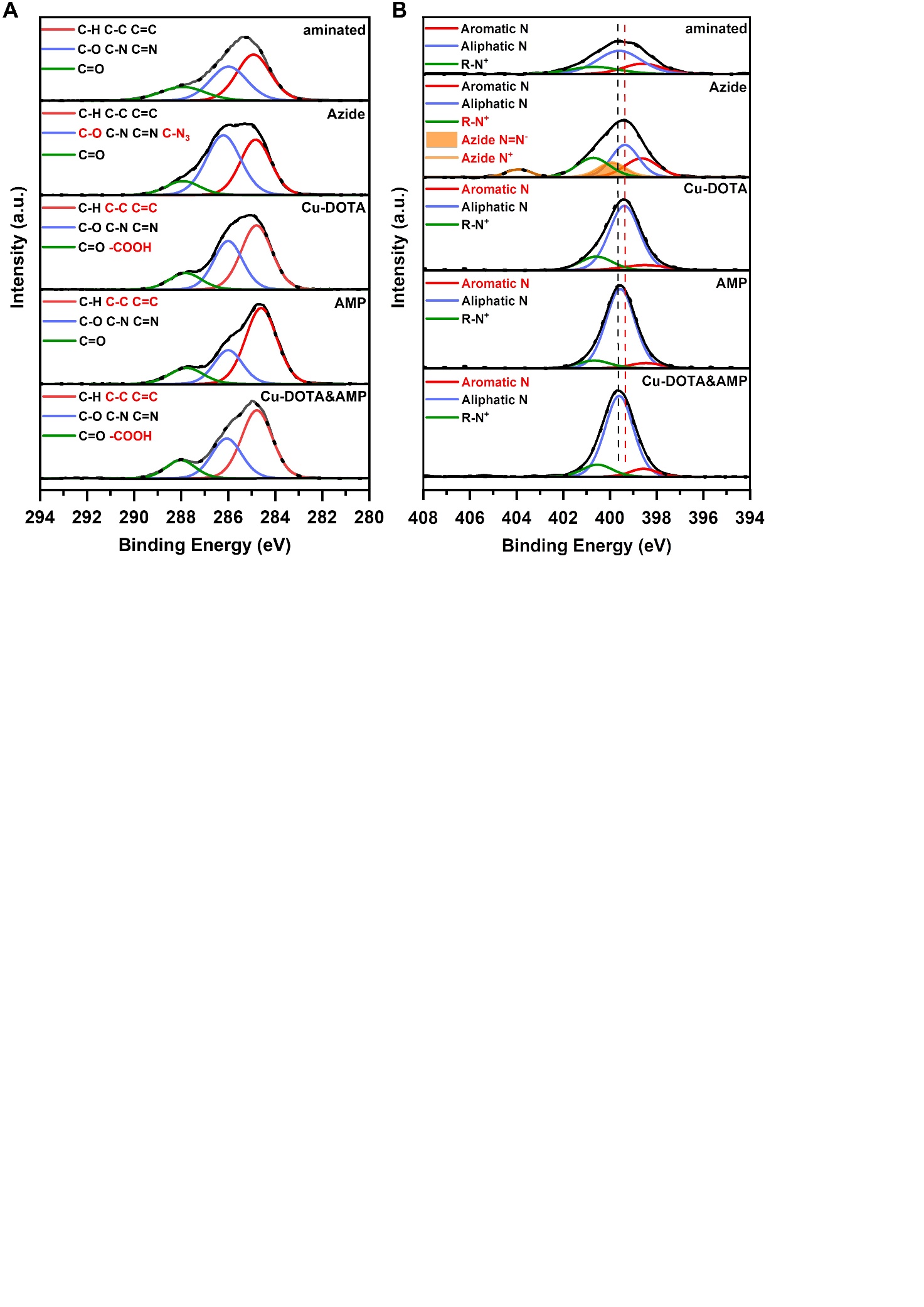


**Figure. S3.** **High resolution spectra** of (**A**) C1s signal on the Azide, AMP, Cu-DOTA, and Cu-DOTA&AMP surfaces. (**B**) N1s signal on the Azide, AMP, Cu-DOTA, and Cu-DOTA&AMP surfaces.


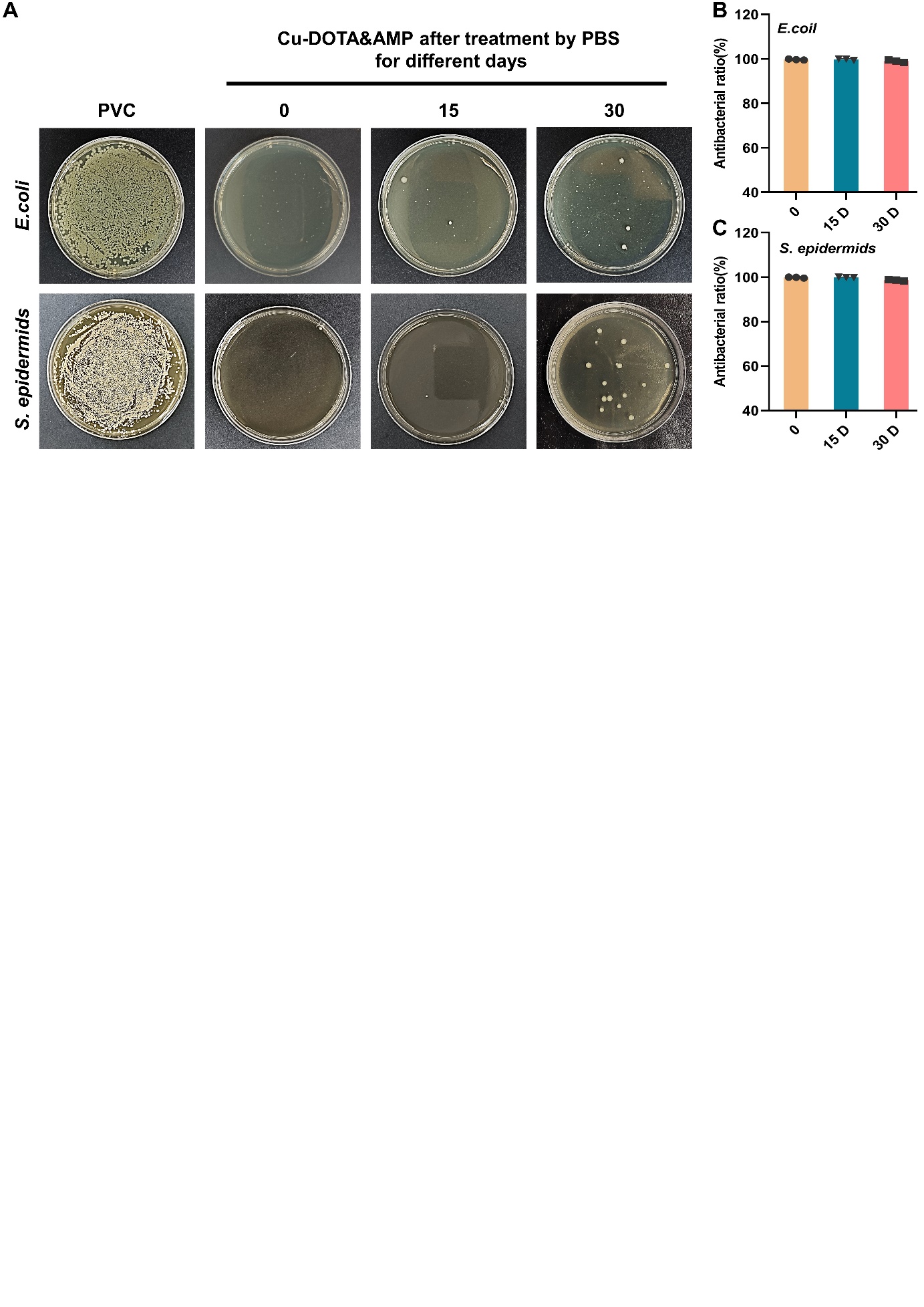


**Figure. S4. Stability of the antibacterial ability of Cu-DOTA&AMP coating.** (**A**) Representative colonization of E. coli and S. epidermids on bare PVC and Cu-DOTA&AMP-coated PVC before and after treatments by PBS for different days. The solution was replaced every 24 h. (**B**) and (**C**) Antibacterial rates, calculated from the results of (**A**). Data are presented as mean ± SD (n=4) and analyzed by one-way ANOVA (*p<0.05, **p < 0.01, ***p < 0.001).


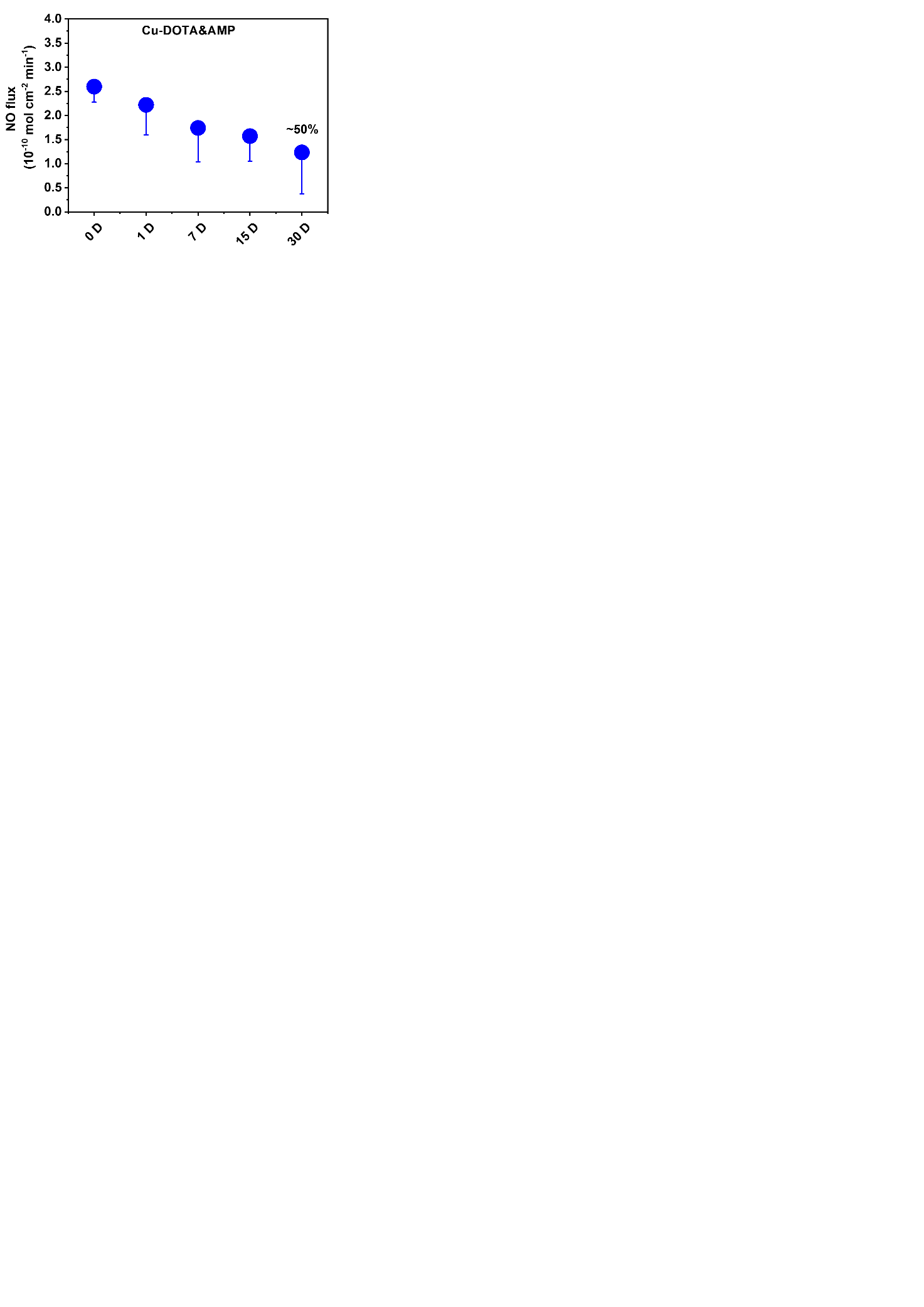


**Figure. S5. Stability of the NO catalytic release ability of Cu-DOTA&AMP coating.** The Cu-DOTA&AMP coating was soaked in PBS buffer and retained the NO catalytic releas rate of 50 percent for 30 days. The solution was replaced every 24 h.


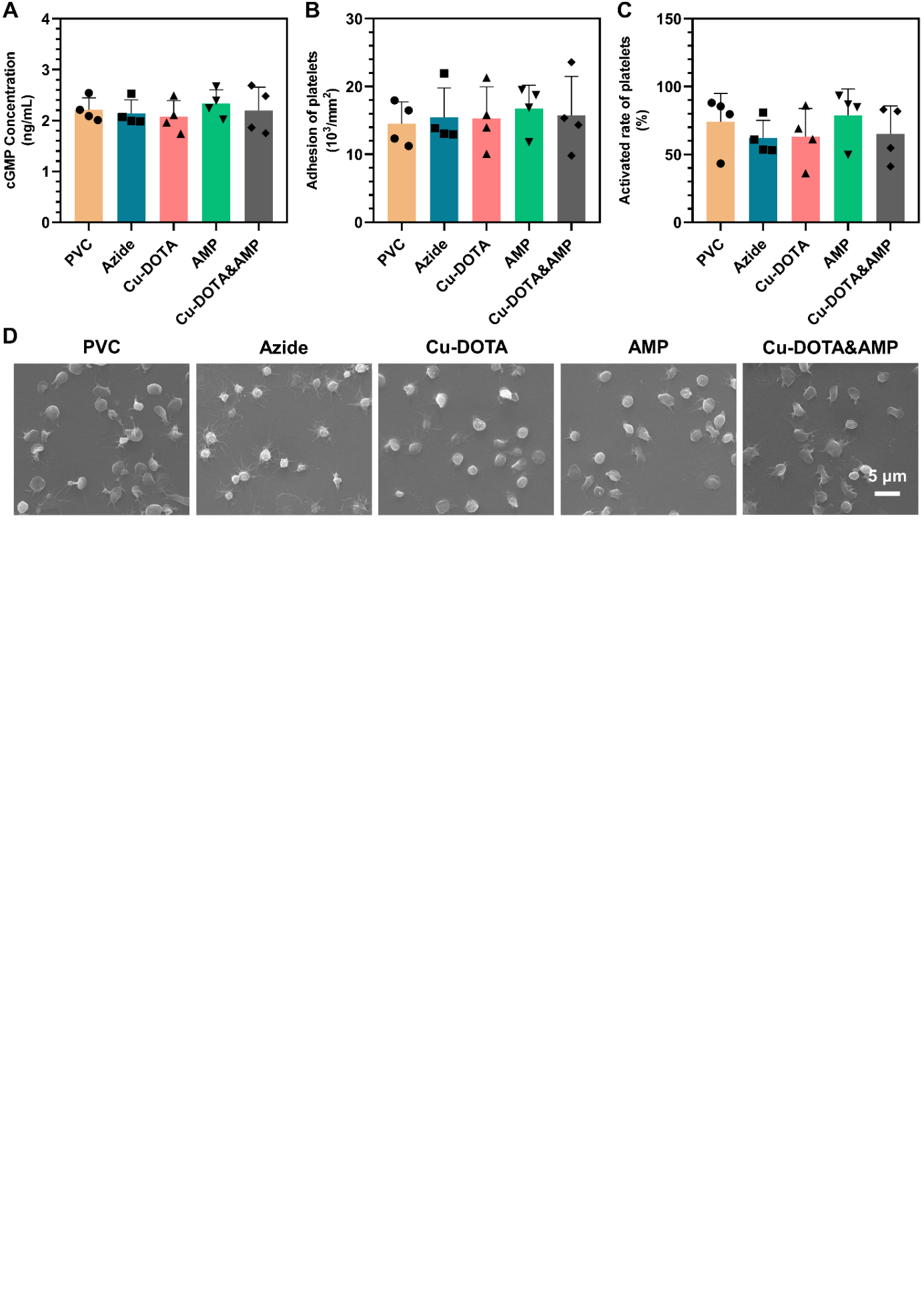


**Figure. S6. In vitro blood compatibility tests without NO donor.** (**A**) Concentration of cGMP synthesized by platelets. (**B**) Adhesion number, (**C**) activation rate of the adhered platelets and (**D**) SEM images on bare and modified PVC. Data presented as mean ± SD and analyzed using a one-way ANOVA, **p < 0.01, ***p < 0.001.

**
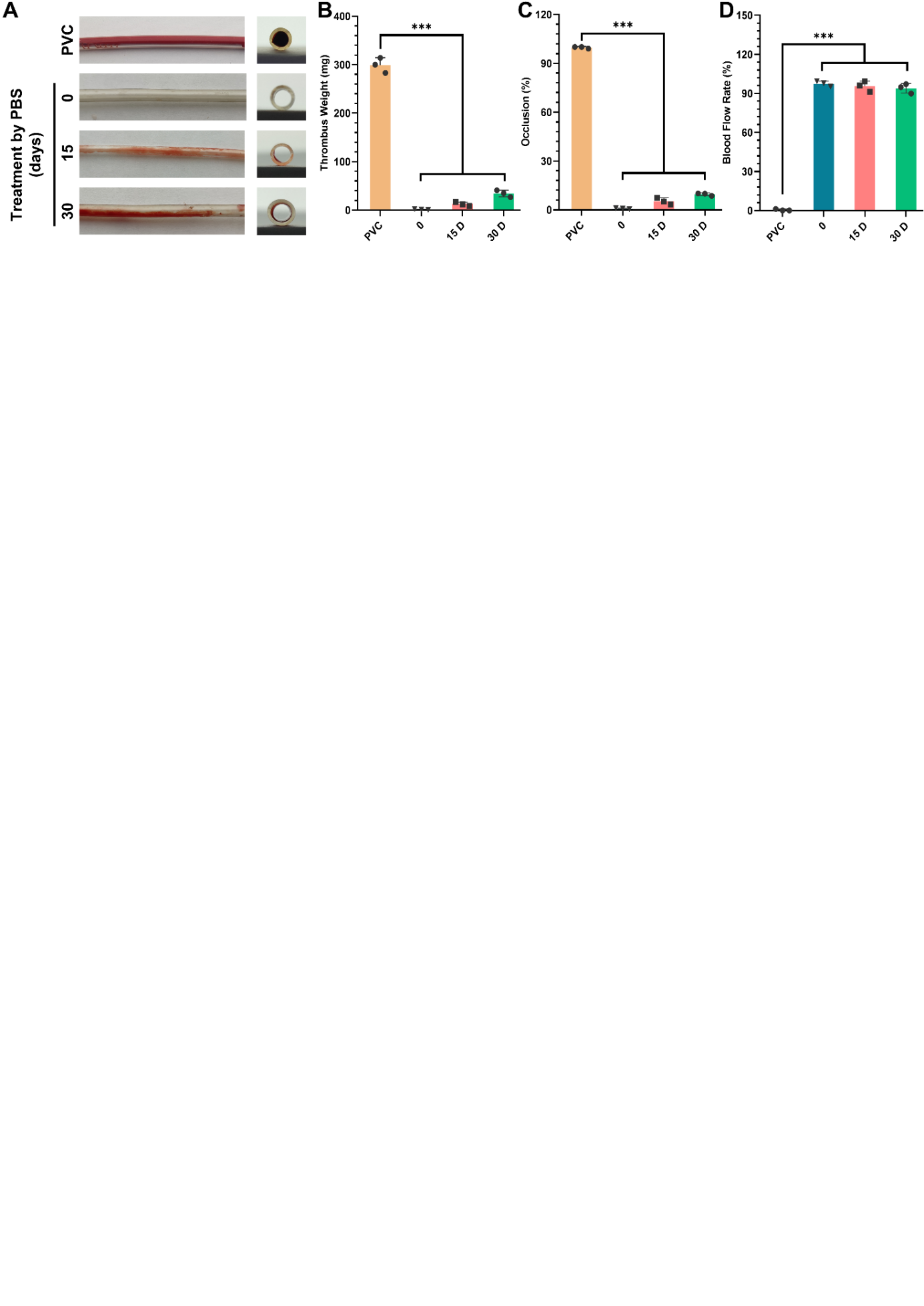
**

**Figure. S7. Stability of the Anticoagulation ability of Cu-DOTA&AMP coating.** (**A**) Photographs and cross-sectional photographs of the unmodified- and Cu-DOTA&AMP-modified circuits before and after treatments by PBS for different days. The solution was replaced every 24 h. Thrombus weight (**B**) and occlusion rates (**C**) of different circuits. (**D**) Blood flow rates in different circuits at the end of circulation experiments. Data are presented as mean ± SD (n=4) and analyzed by one-way ANOVA (*p<0.05, **p < 0.01, ***p < 0.001).

**Table S1.** **The atomic compositions of** **different surfaces.**

| Sample | C (%) | N (%) | O (%) | Cu (%) |
| --- | --- | --- | --- | --- |
| Aminated | 77.9 | 11.5 | 10.6 | – |
| Azide | 66.7 | 13.1 | 20.1 | – |
| Cu-DOTA | 64.9 | 13.3 | 20.2 | 1.6 |
| AMP | 67.4 | 14.2 | 18.3 | – |
| Cu-DOTA&AMP | 66.0 | 14.8 | 18.7 | 0.5 |

**Table S2.** **The high-resolution C1s compositions of different surfaces.**

| Sample | C-H C-C C=C  (%) | C-O C-N C=N C-N_3_  (%) |  | C=O (%) |
| --- | --- | --- | --- | --- |
| Aminated | 45.2 | 36.7 |  | 18.1 |
| Azide | 40.0 | 48.8 |  | 11.2 |
| Cu-DOTA | 50.5 | 35.9 |  | 13.6 |
| AMP | 61.0 | 25.0 |  | 14.0 |
| Cu-DOTA&AMP | 54.6 | 31.2 |  | 14.2 |

**Table S3.** **High-resolution N1s compositions of different surfaces.**

| Sample | Aromatic N (%) | Aliphatic N (%) | Azide ((N)-N=) (%) | R-N^+^ (%) | Azide (-N^+^) (%) |
| --- | --- | --- | --- | --- | --- |
| Aminated | 22.9 | 57.1 | 0.0 | 20 | 0.0 |
| Azide | 22.6 | 32.3 | 14.8 | 23.2 | 7.0 |
| Cu-DOTA | 8.2 | 74.6 | 0.0 | 17.2 | 0.0 |
| AMP | 5.9 | 84.8 | 0.0 | 9.32 | 0.0 |
| Cu-DOTA&AMP | 7.2 | 80.0 | 0.0 | 12.8 | 0.0 |
